# *Caenorhabditis elegans* Operons Contain a Higher Proportion of Genes with Multiple Transcripts and Use 3′ Splice Sites Differentially

**DOI:** 10.1371/journal.pone.0012456

**Published:** 2010-08-27

**Authors:** Fei Wang, Shi Huang, Long Ma

**Affiliations:** State Key Laboratory of Medical Genetics, Central South University, Changsha, China; European Bioinformatics Institute (EBI), United Kingdom

## Abstract

RNA splicing generates multiple transcript isoforms from a single gene and enhances the complexity of eukaryotic gene expression. In some eukaryotes, operon exists as an ancient regulatory mechanism of gene expression that requires strict positional and regulatory relationships among its genes. It remains unknown whether operonic genes generate transcript isoforms in a similar manner as non-operonic genes do, the expression of which is less likely limited by their positions and relationships with surrounding genes. We analyzed the number of transcript isoforms of *Caenorhabditis elegans* operonic genes and found that *C. elegans* operons contain a much higher proportion of genes with multiple transcript isoforms than non-operonic genes do. For genes that express multiple transcript isoforms, there is no apparent difference between the number of isoforms in operonic and non-operonic genes. *C. elegans* operonic genes also have a different preference of the 20 most common 3′ splice sites compared to non-operonic genes. Our analyses suggest that *C. elegans* operons enhance expression complexity by increasing the proportion of genes that express multiple transcript isoforms and maintain splicing efficiency by differential use of common 3′ splice sites.

## Introduction

RNA splicing generates multiple transcript isoforms from a single gene and is believed to be a driving force for biological complexity in evolution [1], [2]. In *C. elegans*, over 13% of genes are alternatively spliced [3]. In human, most genes are alternatively spliced [4], [5], [6]. Compared to RNA splicing, operons provide a different regulatory form of gene expression. An operon is a cluster of genes that are transcribed from a single promoter and controlled by the same regulatory sequences [7]. Operons exist abundantly in prokaryotes and are also found in eukaryotes, which include the nematode *Caenorhabditis elegans*, the fruit fly *Drosophila melanogaster* and some mammals [7], [8]. In *C. elegans*, it was initially estimated that there were 15% of genes in about 1000 operons with an average of 2.8 genes per operon [9], [10]. Recently the number of annotated operons in the *C. elegans* genome has increased to approximately 1250 (Wormbase Release 205), which gives an average of 2.3 genes per operon considering the number of operonic genes remains largely unchanged (around 2880, see the Results). In *C. elegans*, genes in an operon form a closely-spaced cluster with an ∼100 bp intergenic distance [10]. However it is not known how operonic genes increase expression complexity, *e.g.*, by RNA splicing, to adjust to the pressure of evolution and at the same time maintain their positional and regulatory relationships. *C. elegans* has a large number of operonic genes that are alternatively spliced, which provides an interesting model to understand the relationship between operons and RNA splicing.

## Results

**Figure 1:**
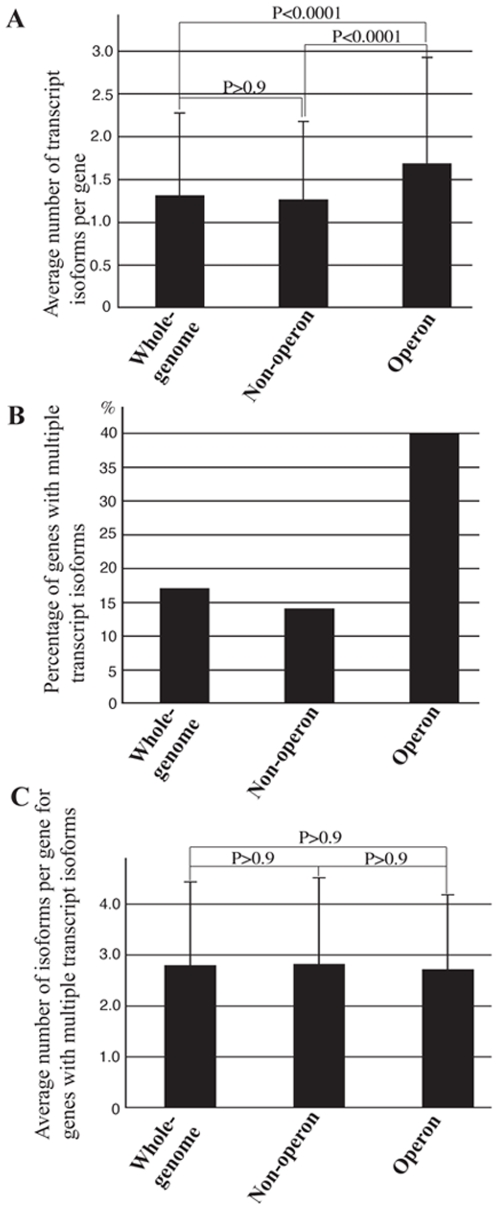
*C. elegans* operons contain a higher proportion of genes that express multiple transcript isoforms. (A) *C. elegans* operonic genes express more transcript isoforms per gene than non-operonic genes do. (B) *C. elegans* operons contain a higher proportion of genes that express multiple transcript isoforms than non-operonic genes do. (C) Alternatively spliced *C. elegans* operonic genes and non-operonic genes have a similar number of transcript isoforms per gene. Z-test was performed (Figure 1A and 1C) to evaluate the significance of difference between the means of transcript numbers. Error bars represent standard deviations.

We examined the average number of transcript isoforms per gene for genes of the whole genome, for all non-operonic genes and for all operonic genes. As shown in Figure 1A, non-operonic genes had about 1.26 transcript isoforms per gene, which was similar to the average of 1.31 transcript isoforms per gene for the whole genome. Operonic genes had 1.68 transcript isoforms per gene, which was over 30% more than that of the non-operonic genes.

One reason that operonic genes have more transcript isoforms per gene than non-operonic genes do is that operons may contain a higher proportion of genes that generate multiple transcript isoforms. Indeed, about 40% of all operonic genes have multiple transcript isoforms (Figure 1B and Table 1). However, only 14% and 17% of non-operonic genes and all genes, respectively, have multiple transcript isoforms (Figure 1B and Table 1). We next examined whether there is any difference in the average number of isoforms for genes that have multiple transcript isoforms. For all such non-operonic genes, there were about 2.81 isoforms per gene. For all such operonic genes, there were 2.71 isoforms (Figure 1C). For all genes of the whole genome, this number was 2.78, which was similar to that of operonic and non-operonic genes (Figure 1C). These results suggest that alternatively spliced operonic and non-operonic genes do not differ apparently in generating transcript isoforms. Therefore, operonic genes may utilize the splicing machinery as efficiently as non-operonic genes do to enhance their expression complexity.

**Table 1:** The numbers of genes and transcripts we analyzed.

	Whole-genome		Non-operon		Operon	
	Genes	Transcripts	Genes	Transcripts	Genes	Transcripts
**Genes with single transcript**	20109	20109	18369	18369	1740	1740
**Genes with multiple transcripts**	4248	11832	3106	8732	1142	3100
**Total**	24357	31941	21475	27101	2882	4840

Genes and annotated transcripts were downloaded from WormMart and processed with MS Excel.

**Figure 2:**
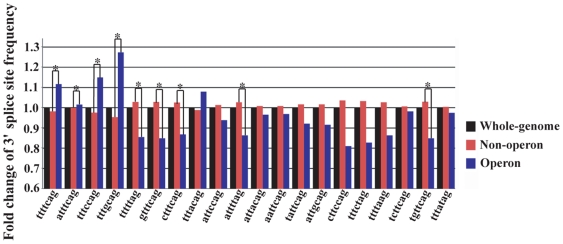
Common 3′ splice sites are used differentially by *C. elegans* operonic genes. The proportions of each 3′ splice site (X axis) of operonic and non-operonic genes were compared to that of all genes of the whole genome and were presented as fold changes (Y axis). Pairwise Z-test was performed (see Table 2) to evaluate the significance of difference between the proportions of each 3′ splice site in operonic genes and non-operonic genes. *: *p*≤0.01.

To investigate whether operonic introns utilize 3′ splice sites differently from non-operonic introns, we analyzed the nucleotide sequences of position −7 to −1 of *C. elegans* introns. This sequence (3′ splice site) is recognized by the splicing factors U2AF large and small subunits and plays important roles in regulating splicing efficiency and alternative splicing [11], [12], [13], [14]. Among all 3′ splice sites, the top 20 most commonly used sites were found in over 80% of introns (Table 2), suggesting that these sites are responsible for the splicing of the majority of introns. As shown in Figure 2, operonic introns use ttttcag, atttcag, tttccag and tttgcag significantly more frequently than non-operonic introns do, in which the frequency of tttgcag usage in operonic introns increased over 30% compared to that in non-operonic introns. 16 sites were used equally or less frequently in operonic introns. Among them, the frequencies of tttttag, gtttcag, ctttcag, attttag and tgttcag were significantly reduced compared to that of non-operonic introns.

**Table 2:** The proportions and numbers of the 20 most frequently used 3′ splice sites in different groups of genes.

	Proportions and numbers of the top 20 3' splice sites in each group of genes						
	Whole-genome		Non-operon		Operon		
3' splice sites	Ratio	n	Ratio	n	Ratio	n	*p*-value
ttttcag	0.261	27890	0.256	23202	0.291	4688	**0**
atttcag	0.14	14919	0.139	12639	0.141	2280	**0.001**
tttccag	0.0857	9149	0.0835	7567	0.0983	1582	**0**
tttgcag	0.0359	3827	0.0341	3094	0.0456	733	**0**
tttttag	0.0346	3695	0.0355	3220	0.0295	475	**0**
gtttcag	0.034	3617	0.0348	3155	0.0287	462	**0**
ctttcag	0.0329	3508	0.0337	3050	0.0285	458	**0.001**
tttacag	0.0295	3152	0.0291	2640	0.0318	512	0.18
attccag	0.0295	3147	0.0298	2703	0.0276	444	0.18
attttag	0.0218	2327	0.0223	2025	0.0188	302	**0.002**
attacag	0.0161	1721	0.0162	1471	0.0155	250	0.37
aattcag	0.0153	1633	0.0154	1395	0.0148	238	0.36
tattcag	0.0137	1457	0.0138	1255	0.0126	202	0.33
attgcag	0.0129	1378	0.0131	1188	0.0118	190	0.04
cttccag	0.0115	1230	0.0119	1080	0.00932	150	0.03
tttctag	0.00949	1012	0.00978	886	0.00783	126	0.02
ttttaag	0.00866	924	0.00887	804	0.00746	120	0.23
tcttcag	0.00838	894	0.00841	762	0.00821	132	0.96
tgttcag	0.008	854	0.00822	745	0.00678	109	**0.01**
tttatag	0.00735	783	0.00737	668	0.00715	115	0.96
**Total**	**0.81628**	**87117**	**0.81085**	**73549**	**0.84225**	**13568**	NA

Total numbers of each 3′ splice sites were calculated as described in Methods. The proportions of the top 20 sites were presented as a percentage of all identified 3′ splice sites in the groups of genes specified. Pairwise Z-test was performed for each 3′ splice site to test the signficance of difference between proportions of operonic and non-operonic genes. *p*≤0.01 is the confidence level.

## Discussion

It is a challenge for operonic genes to increase expression complexity and maintain splicing efficiency while keeping strict positional and regulatory relationships. *C. elegans* operons may achieve these goals by at least two approaches. First, *C. elegans* operons significantly increase the proportion of genes that express multiple transcript isoforms (Figure 1). However, for genes that express multiple transcript isoforms, there is no apparent difference between the number of isoforms in operonic and non-operonic genes. This result suggests that *C. elegans* operons are more permissive for their genes to increase expression complexity by RNA processing than non-operonic genes are. By increasing the proportion of genes that express multiple transcript isoforms, *C. elegans* operons may compensate for a more strict transcriptional regulation and achieve the goal of expression complexity. Alternatively, *C. elegans* operonic genes may be under more pressure evolutionarily to enhance their transcript complexity, *e.g*., in order to perform more complex biological functions. Second, *C. elegans* operonic genes use four of the 20 most abundant 3′ splice sites (ttttcag, atttcag, tttccag and tttgcag) more frequently and use the other 3′ splice sites equally or less frequently (Figure 2). The differential usage of common 3′ splice sites may help maintain efficient splicing of operonic genes, which are often highly expressed and have essential biological functions [9], [10]. The differential usage of common 3′ splice sites by operonic genes is also consistent with the notion that transcription and RNA splicing are coupled processes [1], [2]. Compared to individual genes, it is plausible that the coupling of transcription and splicing of multiple genes in an operon presents a more challenging task for the splicing machinery, which may favor those 3′ splice sites that optimize the splicing process and result in a differential use of common 3′ splice sites by operonic genes.

The expression of transcript isoforms by *C. elegans* operonic genes may also depend on other regulatory mechanisms, *e.g.*, by using different splicing silencers or enhancers and by generating alternative 5′ and 3′ untranslated regions (UTRs). Further analysis of these possibilities will provide a more comprehensive picture about the expression complexity of *C. elegans* operonic genes.

## Methods

We downloaded *C. elegans* gene names and annotated transcripts from the WormMart (WormBase Release 195) as html files. The data were processed using MS Excel to identify genes with different number of transcripts. Non-operonic genes were identified by deducting operonic genes from all genes of the whole genome. A random examination of over 100 operonic genes that are annotated to have multiple transcript isoforms indicates that the isoforms for each gene share at least one coding exon.

The total number of each analyzed 3′ splice site (positions −7 to −1) for the whole genome was obtained from the Intronerator (http://genome-test.cse.ucsc.edu/Intronerator/) [15]. We downloaded 16,087 unique operonic intron sequences from WormMart (WormBase Release 195) and processed the sequences using a software written in the C programming language and Microsoft Excel. Identical 3′ splice sites (positions −7 to −1) are grouped and the proportion of each site is determined. The number of each 3′ splice site for non-operonic genes was obtained by deducting the number of the same site for operonic genes from the number for the whole genome. The online calculator for pairwise Z-test analysis is found at http://www.dimensionresearch.com/resources/calculators/ztest.html.
